# Irreversible photo-Fenton-like triggered agglomeration of ultra-small gold nanoparticles capped with crosslinkable materials†

**DOI:** 10.1039/c8na00353j

**Published:** 2019-04-09

**Authors:** Maurizio Celentano, Anshuman Jakhmola, Paolo Antonio Netti, Raffaele Vecchione

**Affiliations:** Istituto Italiano di Tecnologia, IIT@CRIB Largo Barsanti e Matteucci 53 80125 Napoli Italy m.celentano@qub.ac.uk raffaele.vecchione@iit.it; Centro di Ricerca Interdipartimentale sui Biomateriali CRIB, Universitã di Napoli Federico II Piazzale Tecchio 80 80125 Napoli Italy

## Abstract

A photo-Fenton-like process can promote the agglomeration and LSPR red-shifting of ultra-small gold nanoparticles by triggering a crosslink-degradation pathway that involves the surface coating, Fe(iii)–citrate and hydrogen peroxide. Applications may range from controlled photo-deposition of active materials to asynchronous sensing technologies to light-focused microfabrication.

## Abstract 2

A photo-Fenton-like process triggers the agglomeration of ultra-small gold nanoparticles by virtue of a crosslink-degradation process involving the nanoparticle coating.

## Graphical abstract

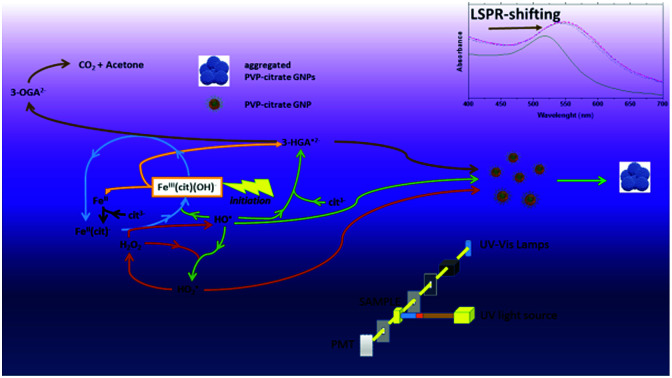

Plasmonic micro- and nano-structures have gained much attention due to their potential as micro- and nano-devices with applications ranging from molecular sensing to photonics.^1,2^ Molecular and chemical sensing devices based on metallic nanoparticles supporting the localized surface plasmon resonance (LSPR) is currently of high interest and improvements in fabrication of micro- and nanostructures made by or containing noble metal nanoparticles have led to advances in the field of LSPR technology.^3–14^ In this context, inorganic nanoparticles dispersed in a polymer matrix with demonstrable applications have been produced by many different *in situ* or *ex situ* approaches.^11–14^ The present work makes use, for the first time, of an Fe(iii)–citrate photo-Fenton-like process to trigger the agglomeration of ultra-small gold nanoparticles (1–3 nm), called AuPVP NPs throughout this paper, leading to LSPR red-shifting. The agglomeration was due to AuPVP NP crosslink-degradation that stems from the photo-Fenton-like process. The rate and degree of the shifting can be modulated by playing with the nanoparticle surface coating and iron(iii) concentration. The whole process results in the formation of a polymer/gold nanoparticle composite.

The Fenton reaction is an advanced oxidative process (AOP) which has been extensively applied in wastewater purification.^15^ In such a process, Fe(ii) is oxidized to Fe(iii) and H_2_O_2_ is reduced to a hydroxide ion and hydroxyl radical (eqn (1)).^16^

1 Fe(ii) + H_2_O_2_ → Fe(iii) + OH˙ + OH^−^, *k* = 76 M^−1^ s^−1^

The irradiation of Fenton's systems (photo-Fenton process) with UV-A and visible light strongly accelerates the reaction rate^17,18^ by virtue of the photochemical reduction of Fe(iii) back to Fe(ii) (eqn (2)).

2 Fe(iii) + H_2_O + *hν* → Fe(ii) + OH˙ + H^+^

This reaction (eqn (2)) results in the generation of additional hydroxyl radicals, which are responsible for degradation and crosslinking of organic materials.

Poly-N-vinylpyrrolidone/citrate-capped gold nanoparticles (AuPVP NPs) with size in the ultra-small size range (1–3 nm) (Fig. 1a, S3a and S4a†), prepared and purified accordingly to a previous protocol,^19^ were subjected to a photo-Fenton-like process at near-neutral pH under anaerobic conditions which in turn results in agglomeration and LSPR red-shifting. The effect of the polymer chain length on LSPR red-shifting was taken into account (later on discussed) using PVPs of several different molecular weights (PVP@10 kDa, PVP@29 kDa and PVP@44 kDa) to synthesize AuPVP NPs. Photo-Fenton conditions were recreated following the procedure reported in the ESI† and the final concentrations and pH were as follows: *C*_Fe(III)_ = 2.0–20.0–80.0 μM; *C*_H_2_O_2__ = 0.8 mM; CNPs = 10 μM; pH = 6.1. The citrate ions, which cap the nanoparticles along with PVP chains,^19^ are excellent chelating agents for the ferric ions^20^ that prevented them from hydrolysis and precipitation even at the near-neutral pH used in this work (pH = 6.1).

**Fig. 1:**
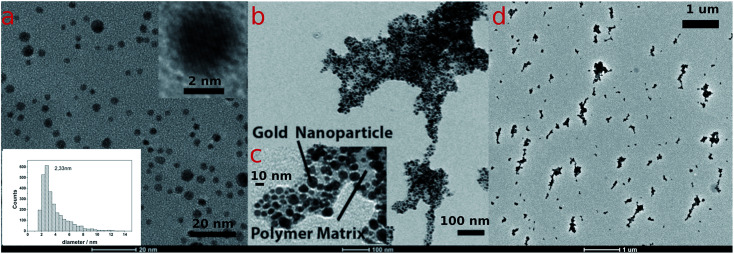
(a) TEM images of AuPVP@10 kDa NPs and size distribution (scale bar 20 nm), insets: size distribution and TEM of a single particle (scale bar 2 nm); (b) sub-micrometric gold nanoparticle agglomerates after irradiation (365 nm at 10W cm^−2^ for 450 s) under photo-Fenton conditions (scale bar 100 nm); (c) TEM analysis showed agglomerates embedded in a low contrast material ascribed to the polymer matrix (scale bar 10 nm); (d) low magnification TEM image of AuPVP@10 kDa NP agglomerates (scale bar 1 μm).

LSPR spectral peaks were measured, before and after UV light irradiation (the detailed experimental set-up is described in the ESI†), for (i) pristine materials (AuPVP NPs), (ii) AuPVP NPs after mixing with either Fe(iii) ions or hydrogen peroxide and (iii) AuPVP NPs under photo-Fenton conditions (mixed with both Fe(iii) ions and hydrogen peroxide).

AuPVP NPs were stable against Fe(iii) ions and hydrogen peroxide when not irradiated (Fig. 2a, data shown for AuPVP@10 kDa NPs) and were also stable against either Fe(iii) ions or hydrogen peroxide when irradiated (Fig. 2b, data shown for AuPVP@10 kDa NPs). Such results further confirmed the extra-stability of the ultra-small AuPVP NPs, as discussed in detail by Celentano *et al.*^19^ In fact, gold nanoparticles were stable even under much harsher conditions such as a strong oxidizing environment due to excess H_2_O_2_, intense UV irradiation and interaction with iron(iii), which are all usually destabilizing.^21–23^

**Fig. 2:**
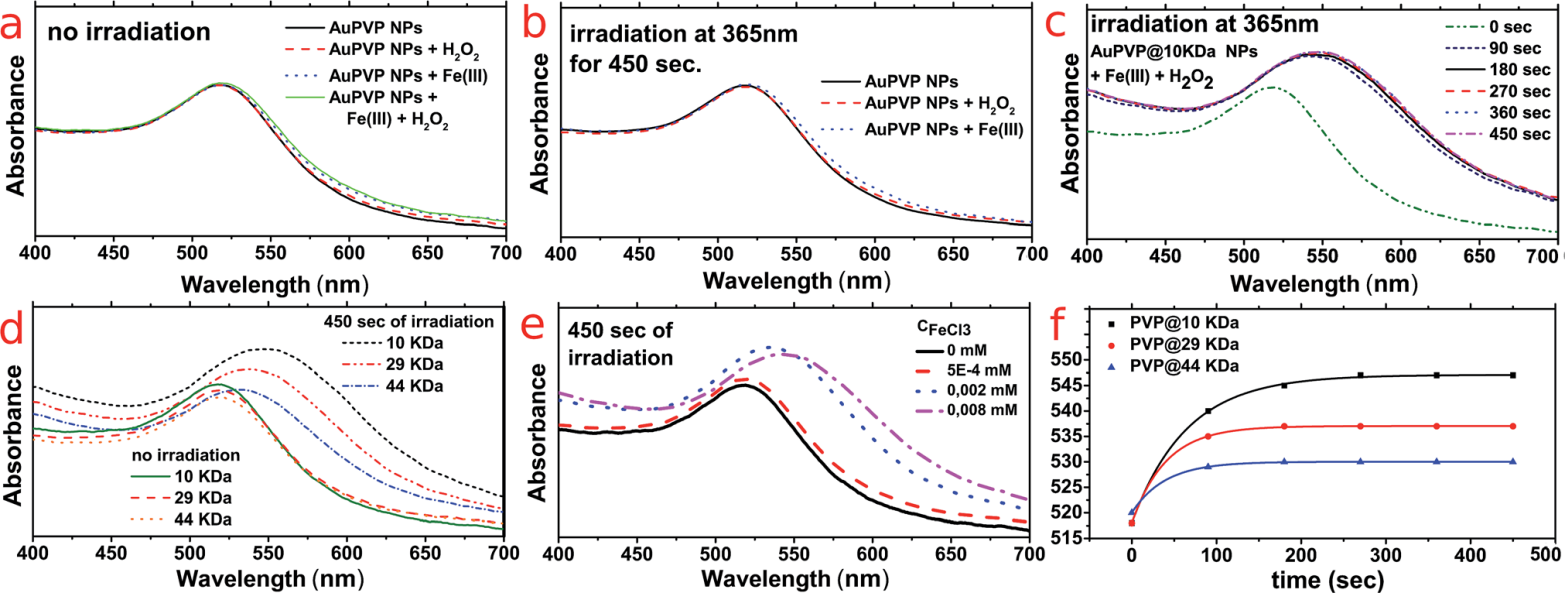
UV-visible spectra of (a) non-irradiated AuPVP@10 kDa NPs taken 450 s after mixing either with Fe(iii) chloride or hydrogen peroxide or both, (b) AuPVP@10 kDa NPs taken after 450 s of irradiation, both as a pristine material and in the presence of either Fe(iii) chloride or hydrogen peroxide, and (c) AuPVP@10 kDa NPs irradiated (365 nm at 10 W cm^−2^ for 450 s) in the presence of Fe(iii) chloride and hydrogen peroxide (*C*_FeCl_2__ = 8.0 × 10^−3^ mM and *C*_H_2_O_2__ = 0.8 mM). Effect of the PVP molecular weight (PVP@10 kDa, @29 kDa, and @44 kDa) and Fe(iii) chloride concentration on LSPR-shifting: (d) LSPR bands of PVP/citrate-coated gold nanoparticles produced using different PVP Mw recorded at 0 and 450 s of irradiation; (e) LSPR bands of AuPVP@10 kDa produced using different Fe(iii) chloride concentrations and recorded after 450 s of irradiation; (f) LSPR-shifting rate as a function of the time and PVP Mw (*C*_FeCl_3__ = 8.0 × 10^−3^ mM). PVP/citrate capped gold nanoparticles were extra stable even in the presence of either Fe(iii) chloride or hydrogen peroxide and under Fenton conditions when non-irradiated.

On the other hand, LSPR bands shifted against time by irradiation under photo-Fenton conditions (Fig. 2c). Such shifting stopped immediately upon switching off the irradiation.

It is remarkable to point out that (i) increasing the molecular weight (chain length) of the PVP decreased the LSPR red-shift (Fig. 2d), (ii) the more the concentration of ferric ions the higher the LSPR shifting (Fig. 2e) and (iii) the LSPR shifting had an exponential rate and it differed according to the PVP molecular weight (Fig. 2f).

Variations in the LSPR spectral band, due to interactions between the nanoparticle surface and molecules from the surrounding, have been used as detectable signals in various processes.^24,25^

Such interactions may also lead to unstable nanoparticles which agglomerate or dissolve bringing about changes in optical properties and LSPR-shifting.^21,25–36^

The UV-visible spectra of agglomerating nanoparticles displayed a broad LSPR band. Theoretical studies found out that the total spectrum of a spherical agglomerate exhibits only one distinct broad peak (at 549 nm) in the wavelength of the experimental peaks.^31^ TEM analysis of irradiated AuPVP NPs under photo-Fenton-like conditions displayed nanoparticle agglomerates on a sub-micrometric scale (Fig. 1b and d, data shown for AuPVP@10 kDa NPs), embedded in a low contrast organic material (Fig. 1c, data shown for AuPVP@10 kDa NPs) and composed of individual gold nanoparticles.

Fe(iii)–citrate complexes have been demonstrated to be excellent Fe(iii)-photocatalysts with high photoreactivity.^37–39^ At near-neutral pH (6.1) implemented in this work, the main Fe(iii)–citrate species were FeOHcit^−^ and Fe_2_(OH)_2_(cit)_2_^2−^, where the former dominated, resulting in the presence of a homogeneous photo-catalyst at the pH studied.^40^ FeOHcit^−^ has been reported to photochemical induce HO˙ and 
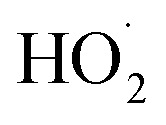
 radicals form H_2_O_2_ and 3-hydroxy glutarate radicals (3-HGA˙^2−^) from citrate ions in aqueous solutions of pH ranging from 3 to 8.^37,41–44^

Despite the photo-reactivity of the Fe(iii)–citrate species, the UV-visible spectroscopy analysis pointed out that the agglomeration was not simply due to photo-generation of 3-HGA˙^2−^ from Fe(iii)–citrate species because both Fe(iii)–citrate and hydrogen peroxide were essential to carry out the process. This point fully validated the photo-Fenton-like nature of the whole process and the photo-Fenton-like reagent was formed by Fe(iii), citrate ions on the gold nanoparticle surface, and hydrogen peroxide. Gold nanoparticles were an integral part of the photo-Fenton-like system.

AuPVP NPs and the agglomerates were further analysed by ATR-FTIR spectroscopy (Fig. 3, data shown for AuPVP@10 kDa NPs) in order to have a deeper understanding of the photo-Fenton-like triggered agglomeration process. Several IR signals faded away and new bands emerged in the ATR-FTIR spectrum (Fig. 3a and b) after UV-irradiation under photo-Fenton-like conditions. Fading of the pyrrolidone ring vibrational modes at 838 (ring breathing), 1291 (C–N stretching), 1370 (CH bending), 1401 (CH bending), 1421 (C–N, CH_2_ scissoring), 1437 (C–N, CH_2_ scissoring), 1462 (C–N, CH_2_ scissoring), 1652 (C=O stretching, amide) and 2950 cm^−1^ (–CH_2_ asymmetric stretching, ring) (Fig. 3b),^45^ along with the emergence of new signals (Fig. 3a) at 1694 (C=O stretching in carboxylic acid) and 1042 cm^−1^ (C–N, aliphatic amine), indicated that radical attacks could open the pyrrolidone rings to form poly(vinyl aminobutyric acid) units.^46,47^ Fading of the signal (Fig. 3b) at 2874 cm^−1^ (–CH_2_ symmetric stretching, chain)^45^ suggested radical abstractions of hydrogen atoms from the polymer chains generating macroradicals. Two macroradical chains might undergo a recombination step to form a covalent bond in a single stable crosslinked polymer. The macroradical might even rearrange to a more stable state by chain scission leading to an increase of the vinyl units. The new signals (Fig. 3a) at 1632 (C=C stretching or N–H amine stretching), 3047 (C–H, alkene) and 3200 cm^−1^ (broad, O–H carboxylic acid) could arise from both pathways.^46^ In addition, it is well established that PVP can be crosslinked by chemical reagents (strong alkali, inorganic persulfates and peroxides) and by gamma or ultraviolet irradiation even under photo-Fenton conditions.^48–50^ Fading of the signal (Fig. 3b) at 1584 cm^−1^ indicated that citrate ions^19^ also reacted and were depleted during photo-Fenton triggered agglomeration. Since ATR-FTIR spectroscopy indicated that the PVP/citrate coating was crosslink-degraded, all the processes described previously might be responsible for the agglomeration of the nanoparticles.

**Fig. 3:**
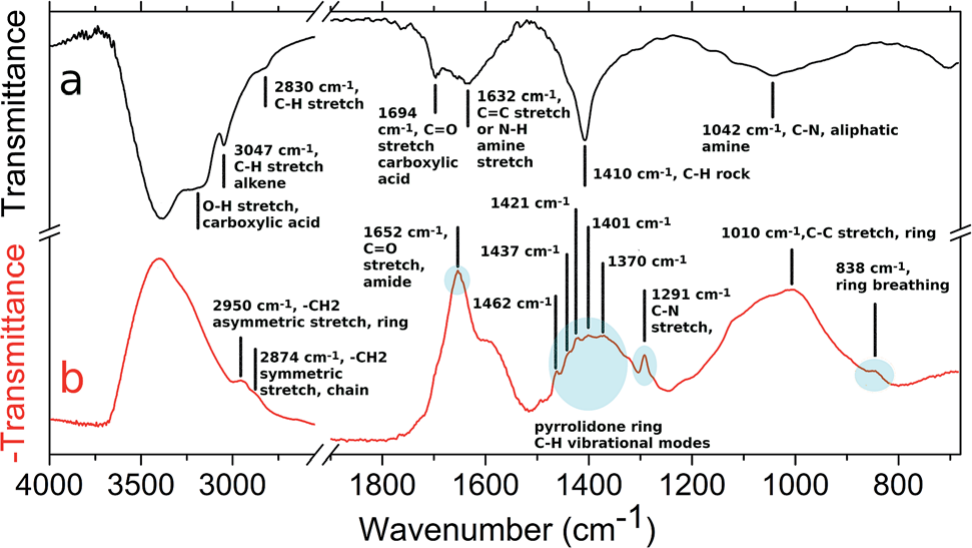
ATR-FTIR spectra of (a) photo-Fenton agglomerated gold nanoparticles (irradiation time 450 s) and (b) AuPVP@10 kDa NPs. Significant vibrational modes are assigned in the graph. Bands with significant signal fading are highlighted.

Based on the results presented here and on the up to date knowledge about the Fe(iii)/citrate photo-Fenton-like system, the schematic in Fig. 4 describes the iron cycling and the main reactions that might be involved in the photo-Fenton-like triggered agglomeration of AuPVP NPs.

**Fig. 4:**
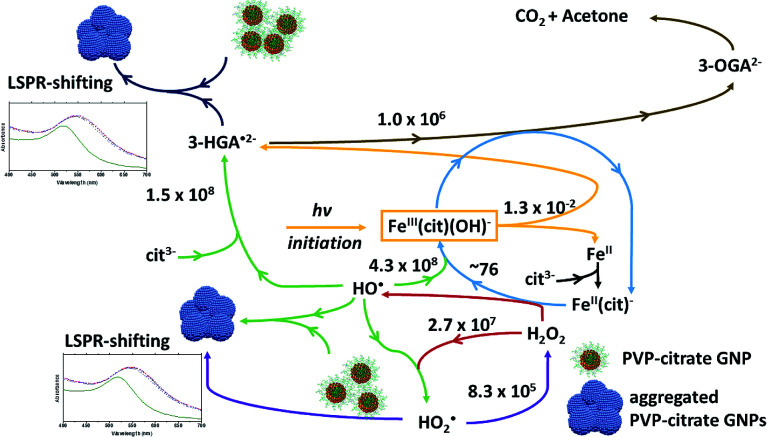
Scheme for the iron cycling and main reactions in ferric–PVP/citrate coated GNP agglomeration systems. Rate constants (M^−1^ s^−1^) from ref. 33. 3-HGA˙^2−^ = 3-hydroxo-glutarate radical, 3-OGA^2−^ = 3-oxo-glutarate, and cit^3−^ = citrate ion.

In conclusion, the main aim of this study was to demonstrate that ultra-small AuNPs, properly coated with PVP and citrate, can be integrated with a well-known advanced oxidative process (photo-Fenton process), generally used for wastewater treatment, resulting in a composite system which displays new features. In this study, we have demonstrated, for the first time, that a simple colloidal gold system could be easily modified into a highly sensitive photo-responsive nanometric system. Indeed, a photo-Fenton-like reaction triggers the agglomeration of PVP/citrate-coated ultra-small gold nanoparticles with consequent LSPR red-shifting. The agglomeration and LSPR-shifting stem from a crosslink-degradation process involving the nanoparticle coating. In fact, the process can be modulated by playing with the nanoparticle surface coating and iron(iii) concentration. These new features open doors for new fields of research involving colloidal gold. For instance, this finding is appealing to a wide general interest readership since it may be of benefit in several applications ranging from light-focused microfabrication, like two-photon-polymerization (2PP) microfabrication, to controlled photo-deposition of active materials supporting LSPR. The system investigated in this work may also find application in asynchronous sensing since each component (gold nanoparticles, ferric ions, and H_2_O_2_ that is a by-product of many enzymatic reactions) is an essential module that once removed from the system prevents its operation. Even though such applications have not been reported in this communication, they should be deemed as a hint to promote scientific discourse that challenges the current state of knowledge in the relative fields.

## Conflicts of interest

There are no conflicts to declare.

## Supplementary Material

NA-001-C8NA00353J-s001
