# Supplementary material for: Irreversible photo-Fenton-like triggered agglomeration of ultra-small gold nanoparticles capped with crosslinkable materials
Source: Nanoscale Adv. 2019 Apr 9;1(6):2146–50. doi: 10.1039/c8na00353j (PMC9418340; doi:10.1039/c8na00353j)
Supplement: NA-001-C8NA00353J-s001 [file NA-001-C8NA00353J-s001.pdf]

Electronic Supplementary Material (ESI) for Nanoscale Advances.
This journal is © The Royal Society of Chemistry 2019

**Supporting Information for**

# Irreversible photo-Fenton-like triggered agglomeration of ultra-small gold nanoparticles capped with crosslinkable materials.

Maurizio Celentano[a*], Anshuman Jakhmola[a], Paolo A. Netti[a,b], Raffaele Vecchione[a,b*]

[a]Istituto Italiano di Tecnologia, IIT@CRIB, Largo Barsanti e Matteucci, 53 80125 Napoli, Italy
[b]Centro di Ricerca Interdipartimentale sui Biomateriali CRIB, Università di Napoli Federico II, Piazzale Tecchio, 80 80125 Napoli, Italy

email address: m.celentano@qub.ac.uk and raffaele.vecchione@iit.it

## Materials

Gold (III) chloride trihydrate (99.9 %), trisodium citrate dihydrate, polyvinylpyrrolidone (PVP, average MW 10,000, 29000 and 44000 Da), hydrogen peroxide (30% w/v) and ferric chloride hydrate (99.9%) were purchased from Sigma Aldrich and were used as received. Milli-Q water was used in all the experiments.

## Gold nanoparticles synthesis

Poly-N-vynilpirrolidone/citrate-capped gold nanoparticles were prepared accordingly to previous report.[1] Aqueous stock solutions of the starting materials (4mM chloroauric acid, 38.8 mM trisodium citrate and 1% w/w PVP) were prepared and then gently mixed for about 5 s. Three different batches of gold nanoparticles were produced PVP molecular weights were used as capping agent: PVP@10KDa, PVP@29KDa and PVP@44KDa.The molar ratio R= $C_{citrate}/C_{HAuCl4}$ was fixed at 9.7 with final concentrations of $C_{HAuCl4}$ = 2.0 mM, $C_{citrate}$ = 19.4 mM and PVP= 20 µM. The reaction mixture was left undisturbed and reduction process was allowed to proceed at room temperature (298K) for 8-9 h. Poly-N-vinylpirrolidone/citrate-capped gold nanoparticles have been washed with Milli-Q water by ultracentrifugation (150000 rpm, 4x 15 min) to remove any unreacted reagents.

## Characterization

Gold nanoparticles were characterized by ATR-FTIR, TEM and UV-visible Spectroscopy. UV-vis Spectroscopy was performed on J-1500 circular dichroism spectrophotometer from JASCO. 1 cm quartz cells were used to measure the absorption spectrum. IR spectra were recorded on a Nicolet 6700 FTIR spectrophotometer (Thermo Scientific) equipped with an attenuated total reflection (ATR) accessory with a single bounce diamond crystal, a DTGS (deuterated triglycine sulfate) detector, and a standard KBr beam splitter. Samples (2 µL) were placed on diamond crystal and dried before the

transmittance spectrum acquisition with a resolution of 4 $cm^{-1}$. TEM images were obtained using a TECNAI 20 G2: FEI COMPANY (CRYO-TEM-TOMOGRAPHY, Eindhoven) with a camera Eagle 2HS. The images were acquired at 200 kV; camera exposure time: 1 s; size 2048 x 2048.

**Experimental procedure and set-up of the Photo-Fenton-like triggered aggregation of gold nanoparticles.**

Aqueous dispersions of the gold nanoparticles $(C_{NPs} \approx 10\ \mu M, pH = 6.1)$, both as-produced and in photo-Fenton conditions, were irradiated ($365\ nm, 10W/cm^2$, in anaerobic condition) in 1 cm Hellma quartz cuvette for fluorescence (equipped with a screw cap with PTFE-coated silicon septum) using an OmniCure S2000 UV Curing System (Lumen Dynamic) and after several irradiation time UV-Visible spectroscopy was performed on J-1500 circular dichroism spectrophotometer (JASCO) equipped with a PMT detector (163-950 nm), an highly efficient nitrogen purge system and a multi-position Peltier cell-chargers MPTC-513, which permits a 90-degree angle fluorescence-type set-up (experimental set-up in Figure S1). An R2000 Radiamiter was used for measuring and calibrate the output from the OmniCure S2000 UV Curing System. A Keplerian telescope was built (two plano convex lenses from Thorlabs, LA1509 and LA1116), placed in a lens tube and distance 110 mm1) to focalize through the sample cuvette 1 inch of collimated UV radiation beam coming from a zoom collimator (OmniCure Adjustable Spot Collimating Adaptor) attached to the optic fiber (LLG) from the OmniCure S2000 UV Curing System through the sample cuvette. The complete set up was LLG (Omnicure) -> Zoom Collimator -> LA1509 (f=100mm) -> LA1116 (f=10mm). In order to recreate the photo-Fenton conditions, aliquots of freshly prepared acidic water solutions of Fe(III) chloride ($10\ mM, pH\ \approx 4.5$ ) and hydrogen peroxide $(100\ mM)$ were immediately added in this order into gold nanoparticles dispersions up to a final concentrations of $< 2.0 - 20.0 - 80.0\ \mu M >$ and $0.8\ mM$, respectively, then the dispersions were placed into the spectrophotometer (J-1500 JASCO) and kept under stirring ( $1000\ rpm$) with a small magnetic PTFE stirring bar at $T = 298K$. The photo-Fenton-like process was triggered by switching on the UV light irradiation.

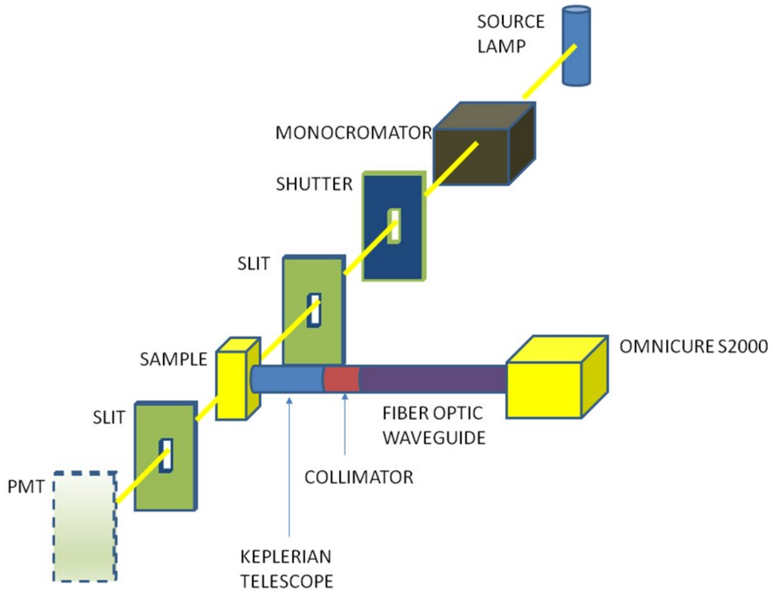


**Figure S1.** Scheme of set-up for UV-Vis measurements with online irradiation.

**UV-Visible spectroscopy of agglomerating gold nanoparticles capped with PVP@29Kda and PVP@44KDa taken during photo-Fenton-like treatment.**

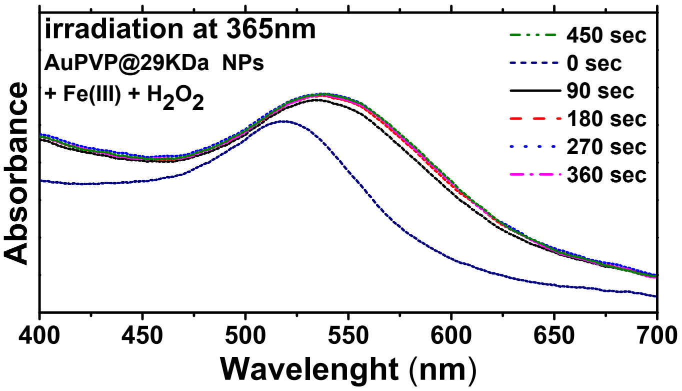


**Figure S1.** UV-Visible spectroscopy of agglomerating gold nanoparticles capped with PVP@29Kda taken during photo-Fenton-like treatment.

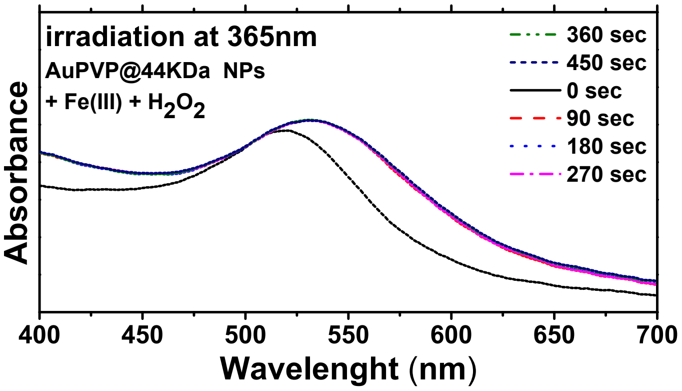


**Figure S2.** UV-Visible spectroscopy of agglomerating gold nanoparticles capped with PVP@44Kda taken during photo-Fenton-like treatment.

**TEM and ATR-FTIR analysis of gold nanoparticles capped with PVP@29Kda and PVP@44KDa taken before and after Photo-Fenton-like triggered aggregation.**

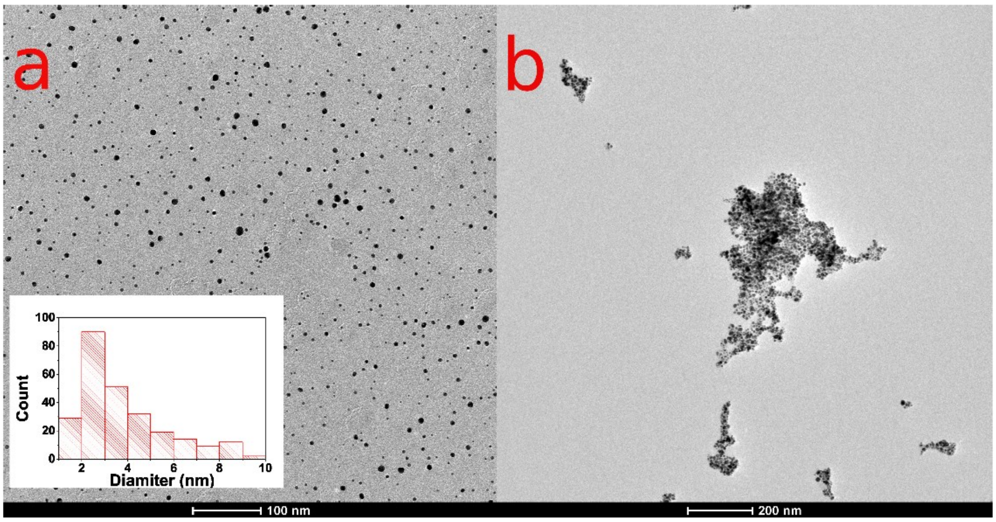


**Figure S3.** TEM micrographs of gold nanoparticles capped with PVP@29Kda before **(a)** and after **(b)** Photo-Fenton-like triggered aggregation.

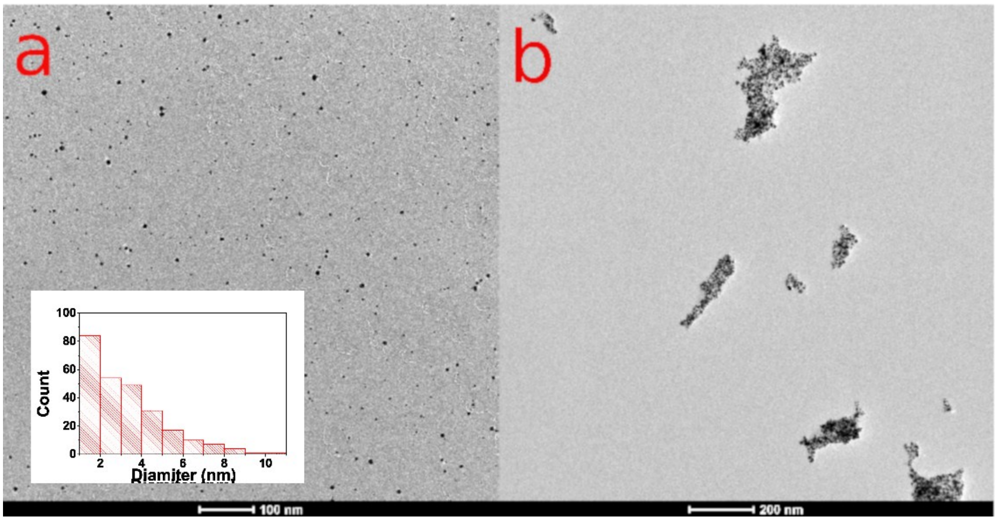


**Figure S4.** TEM micrographs of gold nanoparticles capped with PVP@40Kda before **(a)** and after **(b)** Photo-Fenton-like triggered aggregation.

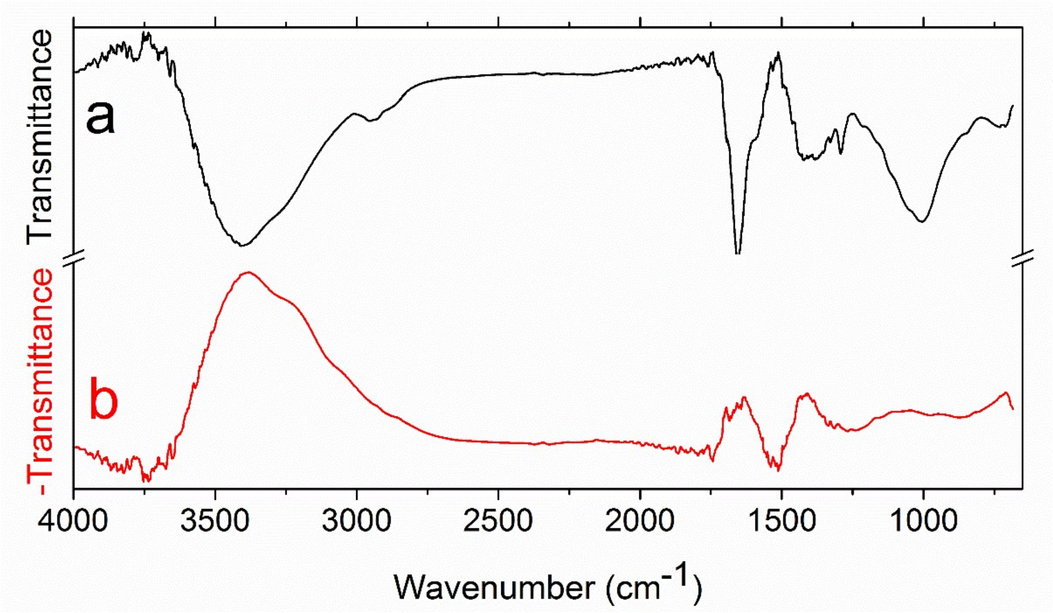


**Figura S5.** *ATR-FTIR* spectra of gold nanoparticles capped with PVP@29Kda before **(a)** and after **(b)** Photo-Fenton-like triggered aggregation.

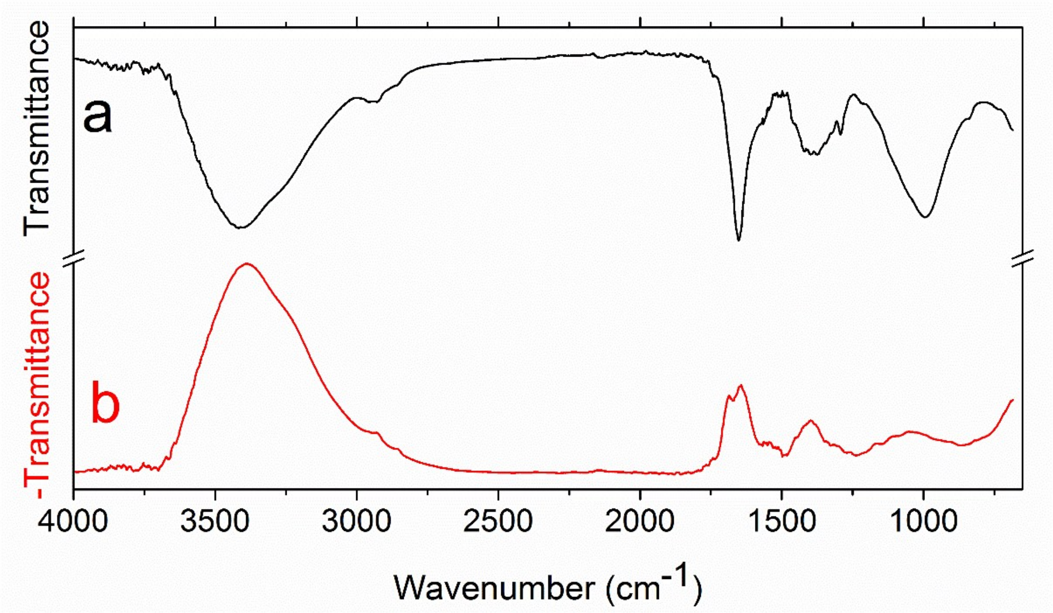


**Figura S6.** *ATR-FTIR* spectra of gold nanoparticles capped with PVP@44Kda before **(a)** and after **(b)** Photo-Fenton-like triggered aggregation.

## References


1. M. Celentano, A. Jakhmola, M. Profeta, E. Battista, D. Guarnieri, F. Gentile, P. A. Netti and R. Vecchione, *Colloids Surfaces A Physicochem. Eng. Asp.*,2018, **558**, 548-557.
